# Human Norovirus Infection in Dogs, Thailand

**DOI:** 10.3201/eid2602.191151

**Published:** 2020-02

**Authors:** Kamonpan Charoenkul, Chanakarn Nasamran, Taveesak Janetanakit, Ratanaporn Tangwangvivat, Napawan Bunpapong, Supanat Boonyapisitsopa, Kamol Suwannakarn, Apiradee Theamboonler, Watchaporn Chuchaona, Yong Poovorawan, Alongkorn Amonsin

**Affiliations:** Chulalongkorn University, Bangkok, Thailand (K. Charoenkul, C. Nasamran, T. Janetanakit, R. Tangwangvivat, N. Bunpapong, S. Boonyapisitsopa, A. Theamboonler, W. Chuchaona, Y. Poovorawan, A. Amonsin);; Mahidol University, Bangkok (K. Suwannakarn)

**Keywords:** norovirus, viruses, human norovirus, canine norovirus, infection, dogs, enteric infections, zoonoses, Thailand

## Abstract

In July 2018, recombinant norovirus GII.Pe-GII.4 Sydney was detected in dogs who had diarrhea in a kennel and in children living on the same premises in Thailand. Whole-genome sequencing and phylogenetic analysis of 4 noroviruses from Thailand showed that the canine norovirus was closely related to human norovirus GII.Pe-GII.4 Sydney, suggesting human-to-canine transmission.

Norovirus infection is a major cause of endemic and epidemic acute gastroenteritis. These viruses have been classified into 7 genogroups on the basis of the major capsid protein, VP1. Noroviruses GI, GII, and GIV can infect humans, GII pigs, GIII and GV ruminants and mice, and GVI and GVII dogs (*1*). The evolutionary mechanism and typing of noroviruses can be analyzed on the basis of recombination between the genes for RNA-dependent RNA polymerase and VP1 (*2*). Newly emerged norovirus strains might lead to increasing incidence of infection worldwide (*3*). The predominant genotype of noroviruses in humans is GII.4. Genetic diversity of noroviruses has been reported in a wide range of animals (e.g., pigs, cattle, and dogs).

In 2007, canine noroviruses in Italy were reported to have the GIV.2 genotype (*4*). Subsequently, these viruses have been reported to cause diseases in dogs in Asia and Europe (*5*–*8*). The seroprevalence of human noroviruses in dogs in the United Kingdom was reported to be 13% (*6*). The GII.4 genotype (variants GII.4-2006b and GII.4-2008) was reported in dogs in Finland, indicating that human noroviruses could be transmitted to and cause diarrhea in dogs (*9*). In humans, antibodies against canine norovirus were also reported in veterinarians, who experienced high risk of exposure (*10*). However, only a few reports describe human norovirus infections in dogs, and limited numbers of complete genomes of canine noroviruses are available in GenBank. We report evidence of human norovirus infection in dogs from a kennel and children on the same premises in Thailand.

## The Study

On July 27, 2018, we investigated acute gastroenteritis in dogs in a dog kennel. An outbreak occurred in a small-scale dog kennel that contained 18 adult dogs in Suphanburi, central Thailand. Clinical signs in bitches and puppies were fever, acute watery diarrhea, and mild dehydration (Appendix Figure 1). Information for the outbreak investigation indicated that 2 weeks earlier (July 18), 2 children (8 months and 2 years of age) who lived on the kennel premises were hospitalized because of vomiting and watery diarrhea. These children recovered within 1 week. During hospitalization, human cases were diagnosed and confirmed as norovirus infection by using a rapid test kit (RIDA QUICK Norovirus, https://clinical.r-biopharm.com). Five adults, 2 children, and 18 adult dogs were living on the premises. All dogs were housed in the kennel; only 2 apparently pregnant dogs (CU21939 and CU21952) were moved into the house of the owner. The 2 apparently pregnant dogs were kept in close contact with children.

On August 2, 2018, a pregnant dog gave birth to 6 puppies, and the other bitch was found to have a false pregnancy. During the 6 weeks (July 27–September 5) of the norovirus outbreak, 2 (11.11%) of 18 dogs (the 2 apparently pregnant dogs kept in the house of the owner) and 5 (83.33%) of 6 puppies showed clinical signs of infection (Appendix Table 1). After treatment and hygiene management, including separation of dogs, frequent cleaning, and disinfection, all dogs recovered, and no deaths occurred.

Animal samples were collected and examined at the Center of Excellence for Emerging and Re-emerging Infectious Diseases in Animals, Chulalongkorn University (Bangkok, Thailand). Studies were approved by the Institutional **Animal Care and Use Committee (approval** no. 1731074). Human samples were collected and submitted to the Center of Excellence for Clinical Virology under the institutional review board of Chulalongkorn University (Institutional Review Board no. 634/59).

During the 4 visits in the study, we examined 75 samples (4 stool samples from 2 children, 71 rectal swab specimens from 18 adult dogs and 6 puppies). We detected norovirus by using a reverse transcription PCR specific for the RNA-dependent RNA polymerase gene as described (*11*,*12*) (Appendix). We detected norovirus in samples from children (4/4), adult dogs (2/53), and puppies (10/18) (Appendix Table 1). All human samples were positive for norovirus at the first (July 27) and third (August 25) visits. The 2 bitches with clinical signs were positive for norovirus at the first visit (July 27). Their puppies (5/6) were positive at the second (August 18) and third (August 25) visits. Our findings are consistent with a previous report that animals can shed noroviruses for a long period (*4*). All samples were also tested for canine parvovirus type 2, rotavirus A, canine coronavirus, and canine distemper virus to rule out other canine enteric diseases; all showed negative results (Appendix Table 1).

**Figure 1:**
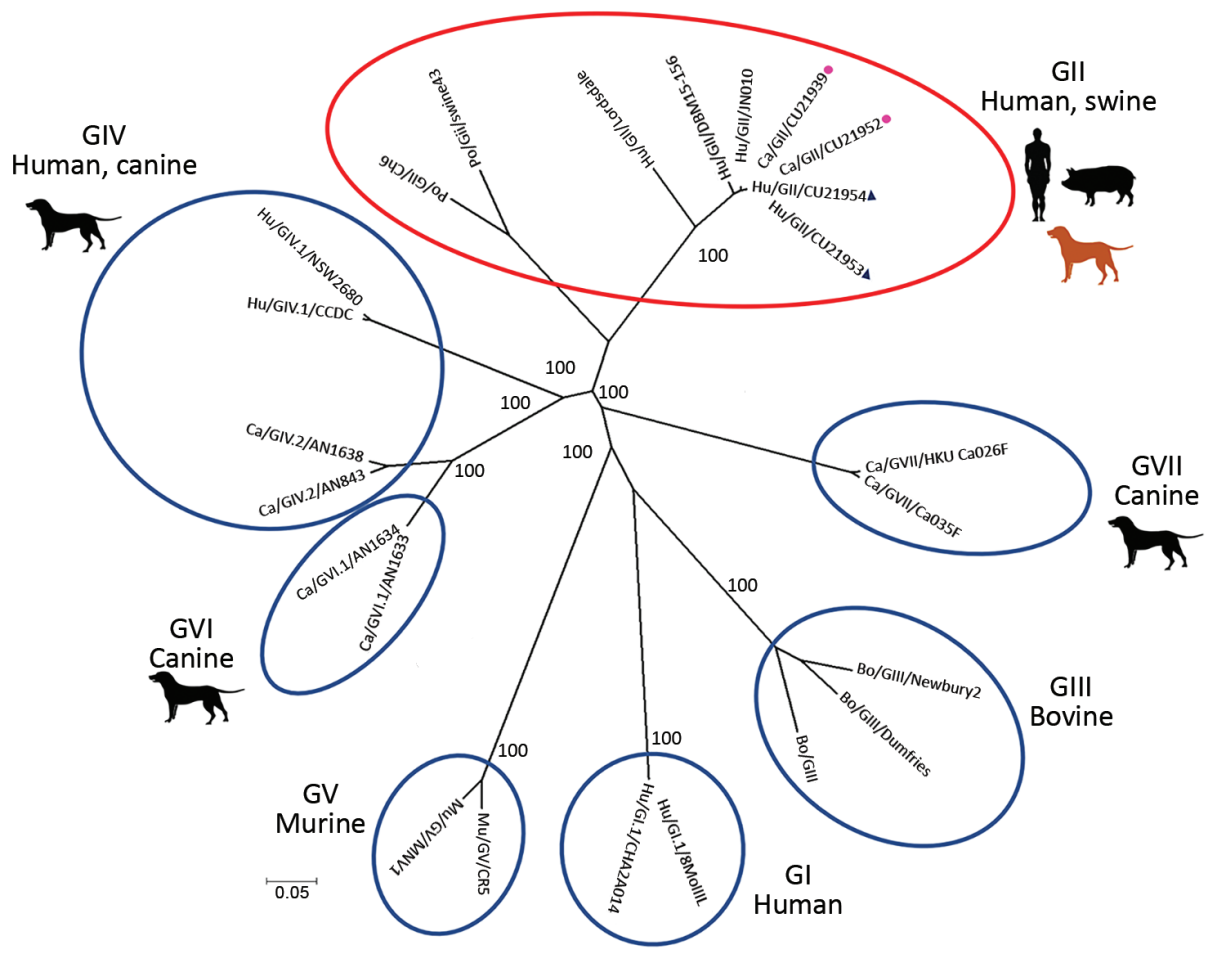
Phylogenetic tree of whole-genome sequences of canine noroviruses (red dots) and human noroviruses (blue triangles) from Thailand and reference sequences. Genogroups GI–GVII are indicated by red oval and blue ovals. The tree was constructed by using MEGA version 7.026 (https://www.megasoftware.net) with the neighbor-joining algorithm and bootstrap analysis with 1,000 replications. Numbers along branches are bootstrap values. Scale bar indicates nucleotide substitutions per site.

We selected 4 of the noroviruses, 2 from humans (CU21953 and CU21954) and 2 from dogs (CU21939 and CU21952), for whole-genome sequencing by using oligonucleotide primer sets (Appendix). We then submitted nucleotide sequences for these viruses (GenBank accession nos. MK928496–9) (Table). Phylogenetic analysis showed that the noroviruses in this investigation clustered in genotype GII.4. In genereal, anine noroviruses are commonly grouped into genogroups GIV, GVI, and GVII. In contrast, noroviruses from these dogs were closely related to human noroviruses and viruses in genogroup GII (Figure 1).

**Table:** Characteristics of noroviruses from humans and dogs, Thailand, July 2018*

Virus	Host	Sample	Age	GenBank accession no.
GII/Hu/THA/2018/GII.Pe-GII.4/CU21953	Human	Feces	2 y	MK928496
GII/Hu/THA/2018/GII.Pe-GII.4/CU21954	Human	Feces	8 mo	MK928497
GII/Ca/THA/2018/GII.Pe-GII.4/CU21939	Dog	Rectal swab	2 y	MK928498
GII/Ca/THA/2018/GII.Pe-GII.4/CU21952	Dog	Rectal swab	3 y	MK928499

*Whole-genome sequences were tested for all isolates.

**Figure 2:**
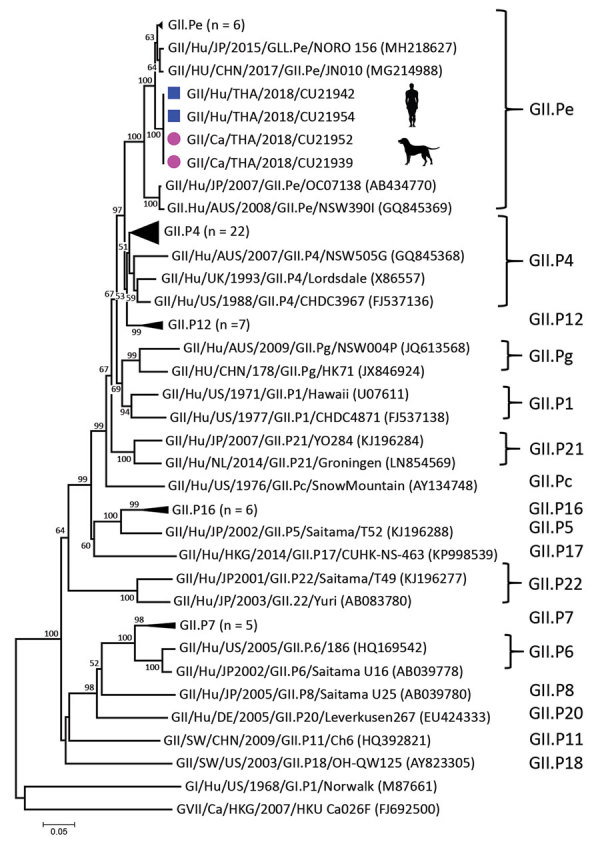
Phylogenetic tree of open reading frame 1 of canine noroviruses (purple dots) and human noroviruses (blue squares) from Thailand and reference sequences. Tree was constructed by using MEGA version 7.026 (https://www.megasoftware.net) with the neighbor-joining algorithm and bootstrap analysis with 1,000 replications. Numbers along branches are bootstrap values, and numbers on the right indicate genogroups. Scale bar indicates nucleotide substitutions per site.

Phylogenetic analysis of partial open reading frame 1 (ORF1) and ORF2 showed that all noroviruses from this investigation clustered with norovirus GII.Pe-GII.4 Sydney 2012, which were reported to be circulating worldwide (Figure 2; Appendix Figure 2) (*3*). Noroviruses from dogs in this study (GII.4 Sydney) were in different clusters from canine noroviruses 3–09 (GII.4 DenHaag) and 261–10 and 1C-09 (GII.4 unclassified) reported in Finland (*9*).

We compared nucleotide and deduced amino acids of the noroviruses from this investigation with reference canine and human noroviruses. On the basis of antigenic epitopes (A–E) of major capsid protein that correlate with blockade of neutralization antibodies, the noroviruses from Thailand had specific amino acids in specific positions consistent with those for human norovirus GII.Pe-GII.4 Sydney, which were not observed in human norovirus genogroups GI and GIV and canine norovirus genogroups GIV and GVII (Appendix Table 2).

Pairwise comparisons of whole-genome sequences showed that the viruses had 99.90% nt identities (only 3 nt differences in ORF2; T1176C [silent mutation 392G], C1354T [silent mutation 452L] and in ORF3; T803A [V268E] to each other and highest nucleotide identities to human norovirus from China [99.00%; JN010] and the human norovirus reference Sydney strain [97.6%; NSW0514]). On the basis of partial ORF2 sequences, we showed that the canine noroviruses from this investigation were different from canine noroviruses GII.4 (3-09, 1C-09, and 261-10; 91.6% nt identities) and GIV, GVI, and GVII (52.90%–55.50% nt identities) (Appendix Table 3).

## Conclusions

We report infection of dogs with human norovirus GII.4 Sydney. Human noroviruses have been reported in dogs in Finland (GII.4 Denhaag and GII.4 unclassified) (*9*). Dogs showed mild clinical signs of acute watery diarrhea, similar to that for human norovirus infection, and low levels of illness and death. Similar observations have also been reported in other studies (*8*,*13*). In this study, children had been hospitalized 2 weeks before the investigation. Disease developed in dogs and puppies after they shared the same premises and possible direct contact with the children. This observation suggests potential human-to-dog transmission of human noroviruses. Genetic and phylogenetic analyses confirmed that whole genomes of canine and human noroviruses were closely related to human norovirus GII.Pe-GII.4 Sydney, suggesting that a common strain is circulating in Thailand and worldwide (*14*,*15*). However, in our study, it is not clear how and when the viruses were introduced to children and dogs.

In summary, we demonstrated evidence of norovirus GII.Pe-GII.4 infection in humans and dogs in Thailand. Dog owners and veterinarians should pay more attention to norovirus infection as a potential zoonotic and reverse zoonotic disease in households, animal hospitals, and shelters. Expanded surveillance for norovirus is needed to determine its status and distribution in human and dog populations.

Appendix Additional information on human norovirus infection in dogs, Thailand.
