# Supplementary material for: Human Norovirus Infection in Dogs, Thailand
Source: Emerg Infect Dis. 2020 Feb;26(2):350–3. doi: 10.3201/eid2602.191151 (PMC6986825; doi:10.3201/eid2602.191151)
Supplement: Appendix — Additional information on human norovirus infection in dogs, Thailand. [file 19-1151-Techapp-s1.pdf]

Article DOI: https://doi.org/10.3201/eid2602.191151

Human Norovirus Infection in Dogs, Thailand

Appendix

Infection in Dogs

During July–September 2018, the Center of Excellence for Emerging and Re-emerging Diseases in Animals at Chulalongkorn University (Bangkok, Thailand) investigated a suspected outbreak of norovirus infection in dogs that had fever, acute vomiting, and watery diarrhea in a small-scale dog kennel. Epidemiologic investigation, sample collection, and laboratory diagnosis were conducted to determine the cause of the outbreak. Information from the outbreak investigation showed that 2 weeks before reporting of cases in animals, 2 children (8 months and 2 years of age) who lived on the kennel premises had been hospitalized on July 18, 2018 because of vomiting and watery diarrhea. These children recovered within 1 week. During hospitalization, human cases were diagnosed and confirmed as norovirus infection by using a rapid test kit. Animal sample collection and testing were performed under the Chulalongkorn University Animal Care and Use Committee Protocol (Institutional Animal Care and Use Committee no. 1731074). Human sample collection and testing were performed at the Center of Excellence for Clinical Virology under the Institutional Review Board of Chulalongkorn University Hospital protocol for human study (Institutional Review Board no. 634/59).

Identification of Viruses

Over 4 visits during July–September 2018, we collected 75 samples: 4 stool samples from 2 children (8 months and 2 years of age) and 71 rectal swab samples from 18 adult dogs and 6 puppies. We identified noroviruses by using an RT-PCR specific for the RNA dependent RNA polymerase gene (*1*,*2*). Because dogs showed clinical signs similar to those for canine enteric diseases, all samples were also examined for canine parvovirus type 2, rotavirus A, canine coronavirus, and canine distemper to rule out other canine enteric diseases (*3*–*7*). We extracted virus RNAs from 10% stool suspensions in phosphate-buffered saline, pH 7.2, and from rectal swab samples by using the QIAsymphony DSP Viral/Pathogen Mini Kit (QIAGEN,

https://www.qiagen.com) following the manufacturer's instructions. The virus RNA was stored at −80°C until use.

A PCR for norovirus identification was conducted as described (*1*,*2*). We use a set of oligonucleotide primers (Appendix Table 4). A 1-step reverse transcription PCR (RT-PCR) (Invitrogen, https://www.thermofisher.com) was conducted in a final volume of 25 μL containing 3 μL of template RNA, 12.5 μL of 2× reaction mixture, 0.6 μL of 10 μmol/L of forward (F4895) and reverse (R5591) primers, 1.2 μL of SuperScript III reverse transcriptase (Invitrogen), and distilled water. The RT-PCR procedure included a reverse transcription step at 55°C for 30 min; an initial denaturation step at 94°C for 2 min; followed by 40 cycles of denaturation at 94°C for 30 s, annealing at 50°C for 30 s, and extension at 68°C for 1 min; and final extension step at 68°C for 6 min. To confirm the presence of noroviruses, 4 μL PCR product was subjected to electrophoresis on a 1.5% agarose gel, with RedSafe dye (Bulldog Bio, https://www.bulldog-bio.com), at 100 V for 45 min. The amplification product was visualized on a UV transilluminator. The expected size of the norovirus-positive amplified product was 493 bp.

We conducted a 1-step real-time RT-PCR for norovirus identification as described (*8*,*9*). This real-time RT-PCR was conducted by using the TaqMan Fast Virus 1-step real-time RT-PCR (Thermo Fisher Scientific, https://www.thermofisher.com) with specific primers and probe to GI and GII noroviruses was conducted in a final volume of 25 μL containing 5 μL of template RNA, 1× Master Mix, 0.25 μmol/L GI forward and reverse primers, 0.125 μmol/L of GI-JOE labeled probe, 0.25 μmol/L GII forward and reverse primers, 0.125 μmol/L of GII-FAM labeled probe, and distilled. This real-time RT-PCR included a reverse transcription step at 50°C for 10 min; an enzyme activation step at 95°C for 20 s; followed by 45 cycles of denaturation at 95°C for 3 s and annealing at 60°C for 30 s. A cycle threshold value <40 was considered as indicating GI and GII positive.

Characterization of Viruses

In this study, we selected 4 noroviruses from Thailand: including 2 from humans (CU21953 and CU21954) and 2 from dogs (CU21939 and CU21952) for whole-genome sequencing. Whole norovirus genomes were sequenced by using oligonucleotide primer sets previously described and new primer sets designed with Primer 3 Plus (Appendix Table 4)

(*10*,*11*). A 25 µL RT-PCR mixture contained 3 µL of template RNA, 12.5 µL of 2× reaction mixture, 0.6 µL of 10 µmol/L forward and reverse primers, 1.2 µL of SuperScript III reverse transcriptase, and distilled water. The RT-PCR procedure included a reverse transcription step at 55°C for 30 min; an initial denaturation step at 94°C for 2 min; followed by 40 cycles of denaturation at 94°C for 30 s, annealing at 48–55°C for 30 s, and extension at 68°C for 2 min; and a final extension step at 68°C for 6 min. Amplicons were gel-purified and sequenced (First Base Laboratories, http://www.firstbaselab.com). Nucleotide sequences were assembled and validated by using SeqMan software version 5.03 (DNASTAR Inc., https://www.dnastar.com). Whole-genome sequences of noroviruses from Thailand were submitted to GenBank under accession nos. MK928496–9.

For pairwise comparisons and genetic analysis of noroviruses from Thailand, we aligned nucleotide sequences and deduced amino acids of noroviruses with reference noroviruses from GenBank by using MEGA version 7.026 (https://www.megasoftware.net) and MegAlign version 5.03 (DNASTAR Inc.) software. For phylogenetic analysis, we compared complete genome sequences of noroviruses from Thailand with those of reference noroviruses, including genogroups GI (n = 2), GII (n = 5), GIII (n = 3), GIV (n = 4), GV (n = 2), GVI (n = 2), and GVII (n = 2). We analyzed the partial open reading frame 1 of noroviruses from Thailand NoVs by comparison with reference GII noroviruses, including GII.P1 (n = 2; United States), GII.P4 (n = 25; Australia, Japan, Georgia, South Korea, the Netherlands, Taiwan, United Kingdom and United States), GII.P5 (n = 1; Japan), GII.P6 (n = 2; Japan and United States), GII.P7 (n = 5; Japan, the Netherlands, and United States), GII.P8 (n = 1; Japan), GII.P11 (n = 1; China), GII.P12 (n = 7; China, South Korea, and Japan), GII.P16 (n = 6; Germany, Japan, Russia, and United States), GII.P17 (n = 1; Hong Kong), GII.P18 (n = 1; United States), GII.P20 (n = 1; Germany), GII.P22 (n = 2; Japan), GII.P21 (n = 2; Japan and the Netherlands), GII.Pc (n = 1; United States), GII.Pe (n = 10; Australia, China, Japan, and Thailand), GII.Pg (n = 2; Australia and China), and outer group GI.P1 (n = 1; United States). We compared the partial open reading frame 2 ORF2 of noroviruses from Thailand with those of reference of GII noroviruses, including genogroups GII.1 (n = 1; United States), GII.2 (n = 1; United Kingdom), GII.3 (n = 3; Argentina, Canada, and the Netherlands), GII.4 (n = 40; Australia, Canada, China, Finland, Ireland, Japan, Netherlands, Thailand, United Kingdom, and United States), GII.5 (n = 1; United Kingdom), GII.6 (n = 22; China, Japan, Italy, Taiwan, United Kingdom, and United States),

GII.7 (n = 12; Japan, Netherlands, Germany, Italy, United Kingdom, and United States), GII.8 (n = 4; China, the Netherlands, and Russia), GII.9 (n = 1; United States), GII.10 (n = 1; Germany), GII.11 (n = 1; Japan), GII.12 (n = 1; United Kingdom), GII.13 (n = 18; China, Nepal, and United States), GII.14 (n = 14; Germany, Japan, and United States), GII.16 (n = 1; United States), GII.17 (n = 1; United States), GII.18 (n = 1; United States), GII.19 (n = 1; United States), GII.20 (n = 1; Germany), GII.21 (n = 18; Bhutan, China, Cambodia, Hong Kong, India, Iraq, Japan, South Korea, Russia, United Kingdom, and United States), GII.22 (n = 1; Japan), and outer groups; GI (n = 1; United States) and GVII (n = 1; Hong Kong). Phylogenetic analysis was performed using MEGA version 7.026 with the neighbor-joining algorithm and bootstrap analysis of 1,000 replications.

References

1. Phumpholsup T, Chieochansin T, Vongpunsawad S, Vuthitanachot V, Payungporn S, Poovorawan Y. Human norovirus genogroup II recombinants in Thailand, 2009–2014. Arch Virol. 2015;160:2603–9. PubMed https://doi.org/10.1007/s00705-015-2545-5

2. Kojima S, Kageyama T, Fukushi S, Hoshino FB, Shinohara M, Uchida K, et al. Genogroup-specific PCR primers for detection of Norwalk-like viruses. J Virol Methods. 2002;100:107–14. PubMed https://doi.org/10.1016/S0166-0934(01)00404-9

3. Buonavoglia C, Martella V, Pratelli A, Tempesta M, Cavalli A, Buonavoglia D, et al. Evidence for evolution of canine parvovirus type 2 in Italy. J Gen Virol. 2001;82:3021–5. PubMed https://doi.org/10.1099/0022-1317-82-12-3021

4. Herrewegh AA, Smeenk I, Horzinek MC, Rottier PJ, de Groot RJ. Feline coronavirus type II strains 79-1683 and 79-1146 originate from a double recombination between feline coronavirus type I and canine coronavirus. J Virol. 1998;72:4508–14. PubMed

5. Frisk AL, König M, Moritz A, Baumgärtner W. Detection of canine distemper virus nucleoprotein RNA by reverse transcription-PCR using serum, whole blood, and cerebrospinal fluid from dogs with distemper. J Clin Microbiol. 1999;37:3634–43. PubMed

6. Mesquita JR, Barclay L, Nascimento MSJ, Vinjé J. Novel norovirus in dogs with diarrhea. Emerg Infect Dis. 2010;16:980–2. PubMed https://doi.org/10.3201/eid1606.091861

7. Gouvea V, Glass RI, Woods P, Taniguchi K, Clark HF, Forrester B, et al. Polymerase chain reaction amplification and typing of rotavirus nucleic acid from stool specimens. J Clin Microbiol. 1990;28:276–82. PubMed

8. Chuchaona W, Chansaenroj J, Wanlapakorn N, Vongpunsawad S, Poovorawan Y. Recombinant GII.Pe-GII.4 norovirus, Thailand, 2017–2018. Emerg Infect Dis. 2019;25:1612–4. PubMed https://doi.org/10.3201/eid2508.190365

9. Debbink K, Costantini V, Swanstrom J, Agnihothram S, Vinje J, Baric R, et al. Human norovirus detection and production, quantification, and storage of virus-like particles. Curr Protoc Microbiol. 2013;31: 15K 1 1–K1 45.

10. He Y, Jin M, Chen K, Zhang H, Yang H, Zhuo F, et al. Gastroenteritis outbreaks associated with the emergence of the new GII. 4 Sydney norovirus variant during the epidemic of 2012/13 in Shenzhen city, China. PLoS One. 2016;11:e0165880. PubMed https://doi.org/10.1371/journal.pone.0165880

11. Untergasser A, Cutcutache I, Koressaar T, Ye J, Faircloth BC, Remm M, et al. Primer3: new capabilities and interfaces. Nucleic Acids Res. 2012;40:e115. https://doi.org/10.1093/nar/gks596 PubMed

Appendix Table 1. Characteristics of samples collected and examined from a dog kennel, Thailand, 2018*

Sample name	Sample ID	Collection date	Sex	Age	Breed	Sample	Clinical sign	NoV RT-PCR	NoV real-time RT-PCR	CPV2	RVA	CaCoV	CDV
First visit, n = 19													
Human 1	CU21953†	Jul 27	M	2 y	Not applicable	Feces	Soft stool	+	+ (27.3)	–	–	NA	NA
Human 2	CU21954†	Jul 27	M	8 mo	Not applicable	Feces	Soft stool	+	+ (20.5)	–	–	NA	NA
Dog 1	CU21936	Jul 27	F	6 mo	French bulldog	Rectal swab	Asymptomatic	–	–	–	–	+	–
Dog 2	CU21937	Jul 27	M	6 mo	French bulldog	Rectal swab	Asymptomatic	–	–	–	–	–	–
Dog 3	CU21938	Jul 27	F	2 y	French bulldog	Rectal swab	Asymptomatic	–	–	–	–	–	–-
Dog 4	CU21939†	Jul 27	F	1 y	French bulldog	Rectal swab	Watery diarrhea	+	+ (29.7)	–	–	–	–
Dog 5	CU21940	Jul 27	F	3 y	French bulldog	Rectal swab	Asymptomatic	–	–	–	–	–	–
Dog 6	CU21941	Jul 27	F	1 y	French bulldog	Rectal swab	Asymptomatic	–	–	–	–	–	–
Dog 7	CU21942	Jul 27	F	1 y	French bulldog	Rectal swab	Asymptomatic	–	–	–	–	+	–
Dog 8	CU21943	Jul 27	F	1 y	French bulldog	Rectal swab	Asymptomatic	–	–	–	–	+	–
Dog 9	CU21944	Jul 27	F	1 y	French bulldog	Rectal swab	Asymptomatic	–	–	–	–	+	–
Dog 10	CU21945	Jul 27	F	1 y	French bulldog	Rectal swab	Asymptomatic	–	–	–	–	–	–
Dog 11	CU21946	Jul 27	F	1 y	French bulldog	Rectal swab	Asymptomatic	–	–	–	–	+	–
Dog 12	CU21947	Jul 27	F	1 y	French bulldog	Rectal swab	Asymptomatic	–	–	–	–	–	–
Dog 13	CU21948	Jul 27	F	1 y	French bulldog	Rectal swab	Asymptomatic	–	–	–	–	–	–
Dog 14	CU21949	Jul 27	M	1 y	French bulldog	Rectal swab	Asymptomatic	–	–	–	–	–	–
Dog 15	CU21950	Jul 27	M	1 y	French bulldog	Rectal swab	Asymptomatic	–	–	–	–	–	–
Dog 16	CU21951	Jul 27	F	1 y	French bulldog	Rectal swab	Asymptomatic	–	–	–	–	–	–
Dog 17‡	CU21952†	Jul 27	F	3 y	French bulldog	Rectal swab	Watery diarrhea	+	+ (29.6)	–	–	–	–
Second visit, n = 24													
Puppy 1§	CU22011	Aug 18	M	2 wk	French bulldog	Rectal swab	Watery diarrhea	+	+ (30.5)	–	–	–	–
Puppy 2	CU22012	Aug 18	M	2 wk	French bulldog	Rectal swab	Watery diarrhea	+	+ (30.1)	–	–	–	–
Puppy 3	CU22013	Aug 18	F	2 wk	French bulldog	Rectal swab	Watery diarrhea	+	+ (31.4)	–	–	–	–
Puppy 4	CU22014	Aug 18	F	2 wk	French bulldog	Rectal swab	Watery diarrhea	+	+ (30.7)	–	–	–	–
Puppy 5	CU22015	Aug 18	F	2 wk	French bulldog	Rectal swab	Watery diarrhea	+	+ (31.8)	–	–	–	–
Puppy 6	CU22016	Aug 18	F	2 wk	French bulldog	Rectal swab	Watery diarrhea	–	–	–	–	–	–
Dog 1	CU22020	Aug 18	F	6 mo	French bulldog	Rectal swab	Asymptomatic	–	–	–	–	–	–
Dog 2	CU22019	Aug 18	M	6 mo	French bulldog	Rectal swab	Asymptomatic	–	+ (36.0)	–	–	–	–
Dog 3	CU22018	Aug 18	F	2 y	French bulldog	Rectal swab	Asymptomatic	–	–	–	–	–	–
Dog 4	CU22034	Aug 18	F	1 y	French bulldog	Rectal swab	Asymptomatic	–	–	–	–	–	–
Dog 5	CU22022	Aug 18	F	3 y	French bulldog	Rectal swab	Asymptomatic	–	–	–	–	–	–
Dog 6	CU22026	Aug 18	F	1 y	French bulldog	Rectal swab	Asymptomatic	–	–	–	–	–	–
Dog 7	CU22021	Aug 18	F	1 y	French bulldog	Rectal swab	Asymptomatic	–	–	–	–	–	–
Dog 8	CU22025	Aug 18	F	1 y	French bulldog	Rectal swab	Asymptomatic	–	–	–	–	–	–
Dog 9	CU22023	Aug 18	F	1 y	French bulldog	Rectal swab	Asymptomatic	–	–	–	–	–	–
Dog 10	CU22029	Aug 18	F	1 y	French bulldog	Rectal swab	Asymptomatic	–	–	–	–	–	–
Dog 11	CU22030	Aug 18	F	1 y	French bulldog	Rectal swab	Asymptomatic	–	–	–	–	–	–
Dog 12	CU22024	Aug 18	F	1 y	French bulldog	Rectal swab	Asymptomatic	–	–	–	–	–	–
Dog 13	CU22031	Aug 18	F	1 y	French bulldog	Rectal swab	Asymptomatic	–	–	–	–	–	–
Dog 14	CU22028	Aug 18	M	1 y	French bulldog	Rectal swab	Asymptomatic	–	–	–	–	–	–
Dog 15	CU22032	Aug 18	M	1 y	French bulldog	Rectal swab	Asymptomatic	–	–	–	–	–	–
Dog 16	CU22027	Aug 18	F	1 y	French bulldog	Rectal swab	Asymptomatic	–	+ (37.0)	–	–	–	–
Dog 17‡	CU22033	Aug 18	F	3 y	French bulldog	Rectal swab	Asymptomatic	–	–	–	–	–	–
Dog 18	CU22017	Aug 18	F	5 y	Miniature pinscher	Rectal swab	Asymptomatic	–	–	–	–	–	–

Sample name	Sample ID	Collection date	Sex	Age	Breed	Sample	Clinical sign	NoV RT-PCR	NoV real-time RT-PCR	CPV2	RVA	CaCoV	CDV
Third visit, n = 9													
Human 1	CU22080	Aug 25	M	2 y	Not applicable	Feces	Asymptomatic	+	S (40.0)	–	–	NA	NA
Human 2	CU22081	Aug 25	M	8 mo	Not applicable	Feces	Asymptomatic	+	+ (33.4)	–	–	NA	NA
Puppy 1	CU22072	Aug 25	M	3 wk	French bulldog	Rectal swab	Watery diarrhea	+	+ (32.3)	–	–	–	–
Puppy 2	CU22073	Aug 25	M	3 wk	French bulldog	Rectal swab	Watery diarrhea	+	+ (33.2)	–	–	–	–
Puppy 3	CU22074	Aug 25	F	3 wk	French bulldog	Rectal swab	Watery diarrhea	+	+ (31.9)	–	–	–	–
Puppy 4	CU22075	Aug 25	F	3 wk	French bulldog	Rectal swab	Watery diarrhea	+	+ (32.5)	–	–	–	–
Puppy 5	CU22076	Aug 25	F	3 wk	French bulldog	Rectal swab	Watery diarrhea	+	+ (32.5)	–	–	–	–
Puppy 6	CU22078	Aug 25	F	3 wk	French bulldog	Rectal swab	Watery diarrhea	–	–	–	–	–	–
Dog 17*	CU22079	Aug 25	F	3 y	French bulldog	Rectal swab	Asymptomatic	–	–	–	–	–	–
Fourth visit, n = 23													
Puppy 1§	CU22143	Sep 5	M	1 mo	French bulldog	Rectal swab	Asymptomatic	–	–	–	–	–	–
Puppy 2	CU22144	Sep 5	M	1 mo	French bulldog	Rectal swab	Asymptomatic	–	–	–	–	–	–
Puppy 3	CU22145	Sep 5	F	1 mo	French bulldog	Rectal swab	Asymptomatic	–	–	–	–	–	–
Puppy 4	CU22146	Sep 5	F	1 mo	French bulldog	Rectal swab	Asymptomatic	–	–	–	–	–	–
Puppy 5	CU22147	Sep 5	F	1 mo	French bulldog	Rectal swab	Asymptomatic	–	–	–	–	–	–
Puppy 6	CU22148	Sep 5	F	1 mo	French bulldog	Rectal swab	Asymptomatic	–	–	–	–	–	–
Dog 1	CU22151	Sep 5	F	7 mo	French bulldog	Rectal swab	Asymptomatic	–	–	–	–	–	–
Dog 2	CU22150	Sep 5	M	7 mo	French bulldog	Rectal swab	Asymptomatic	–	–	–	–	–	–
Dog 3	CU22153	Sep 5	F	2 y	French bulldog	Rectal swab	Asymptomatic	–	NA	–	–	–	–
Dog 5	CU22155	Sep 5	F	3 y	French bulldog	Rectal swab	Asymptomatic	–	–	–	–	–	–
Dog 6	CU22161	Sep 5	F	1 y	French bulldog	Rectal swab	Asymptomatic	–	NA	–	–	–	–
Dog 7	CU22156	Sep 5	F	1 y	French bulldog	Rectal swab	Asymptomatic	–	–	–	–	–	–
Dog 8	CU22152	Sep 5	F	1 y	French bulldog	Rectal swab	Asymptomatic	–	–	–	–	–	–
Dog 9	CU22157	Sep 5	F	1 y	French bulldog	Rectal swab	Asymptomatic	–	–	–	–	–	–
Dog 10	CU22154	Sep 5	F	1 y	French bulldog	Rectal swab	Asymptomatic	–	–	–	–	–	–
Dog 11	CU22158	Sep 5	F	1 y	French bulldog	Rectal swab	Asymptomatic	–	–	–	–	–	–
Dog 12	CU22163	Sep 5	F	1 y	French bulldog	Rectal swab	Asymptomatic	–	NA	–	–	–	–
Dog 13	CU22164	Sep 5	F	1 y	French bulldog	Rectal swab	Asymptomatic	–	NA	–	–	–	–
Dog 14	CU22149	Sep 5	M	1 y	French bulldog	Rectal swab	Asymptomatic	–	NA	–	–	–	–
Dog 15	CU22159	Sep 5	M	1 y	French bulldog	Rectal swab	Asymptomatic	–	NA	–	–	–	–
Dog 16	CU22162	Sep 5	F	1 y	French bulldog	Rectal swab	Asymptomatic	–	NA	–	–	–	–
Dog 17‡	CU22160	Sep 5	F	3 y	French bulldog	Rectal swab	Asymptomatic	–	NA	–	–	–	–
Dog 18	CU22165	Sep 5	F	5 y	Miniature pinscher	Rectal swab	Asymptomatic	–	NA	–	–	–	–

*Numbers in parentheses are cycle threshold values. CaCoV, canine coronavirus; CDV, canine distemper virus; CPV2, canine parvovirus 2; ID, identification; NA, not available; NoV, norovirus; RT-PCR, reverse transcription PCR; RVA, rotavirus A; +, positive; –, negative.
†Samples were subjected to whole-genome sequencing.
‡Dog 17 was a bitch with 6 puppies.
§Puppies 1–6 were from the same litter of dog 17.

Appendix Table 2. Genetic analysis of nucleotide sequences of canine and human noroviruses from Thailand for antigenic epitopes (A–E) major capsid protein compared with those for other noroviruses*

				Antigenic epitopes															
				A						B		C		D			E		
Virus	County/year	GenBank accession no.	Variant	294	296	297	298	368	372	333	382	340	376	393	394	395	407	412	413
Human																			
Lordsdale	UK/1993	X86557	Bristol 1993	A	S	H	D	T	N	L	K	A	Q	D	–†	H	N	T	G
Camberwell	AU/1994	AF145896	Camberwell 1994	V	S	H	D	T	N	L	K	A	Q	D	–†	H	N	T	G
Farmington Hills	USA/ 2002	AY502023	Farmington Hills 2002	A	T	H	N	N	N	M	K	G	E	N	G	T	S	T	G
Hunter504D/04O	AU/2004	DQ078814	Hunter 2004	A	T	Q	N	S	S	V	R	R	E	S	T	T	D	D	S
CGMH09	TW/2006	JN400607	Den Haag 2006b	A	S	R	N	S	E	V	K	G	E	S	T	T	S	N	V
JB-15	KOR/2015	HQ009513	Apeldoorn 2008	T	S	R	N	A	D	V	K	A	D	N	T	A	S	N	S
New Orleans1805	USA/2009	GU445325	New Orleans 2009	P	S	R	N	A	D	V	K	T	E	S	T	T	S	N	I
NSW0514	AU/2012	JX459908	Sydney 2012	T	S	R	N	E	D	V	K	T	E	G	T	T	S	N	T
JN010	CHN/2017	MG214988	Sydney 2012	T	S	H	N	E	N	M	K	T	E	G	T	T	S	N	T
DBM15–156	THA/2015	MG786781	Sydney 2012	T	S	R	N	E	D	M	K	T	E	S	T	T	S	N	T
HuNoV/CU21953	THA/2018	This study	Sydney 2012	T	S	H	N	E	N	M	K	T	E	G	T	T	S	N	T
HuNoV/CU21954	THA/2018	This study	Sydney 2012	T	S	H	N	E	N	M	K	T	E	G	T	T	S	N	T
Canine																			
CaNoV/CU21952	THA/2018	This study	Sydney 2012	T	S	H	N	E	N	M	K	T	E	G	T	T	S	N	T
CaNoV/CU21939	THA/2018	This study	Sydney 2012	T	S	H	N	E	N	M	K	T	E	G	T	T	S	N	T

*AU, Australia; CaNoV, canine norovirus; HuNoV, human norovirus; KOR, South Korea; THA, Thailand; TW, Taiwan.
†–, Gap at position 394.

Appendix Table 3. Pairwise comparisons of nucleotides and amino acids of canine norovirus CU21939 from Thailand with those of reference noroviruses*

						Nucleotide (amino acid) identity, %			
Virus	Host	Genotype†	Country/year	GenBank accession no.	Variant†	WGS 1–7564‡	ORF1 5–5104‡	ORF2 5085–6707‡	ORF3 6707–7513‡
Canine									
AN843	Dog	GIV.2	USA/2011	MK067289	NA	NA	62.20 (47.80)§	55.30 (38.80)	50.90 (41.40)
170/07	Dog	GIV.2	Italy/2007	EU224456	NA	NA	64.50 (71.20)¶	54.20 (36.50)	51.40 (42.90)
AN1610	Dog	GIV.2	USA/2017	MK067288	NA	NA	62.30 (47.8)§	55.20 (38.10)	51.10 (42.00)
AN1663	Dog	GIV.2	USA/2017	MK067291	NA	NA	62.30 (47.8)§	55.10 (38.30)	51.20 (41.70)
AN1638	Dog	GIV.2	USA/2017	MK067290	NA	NA	62.60 (48.40)§	55.10 (38.30)	51.20 (41.70)
C33/Viseu	Dog	GVI.2	Portugal/2007	GQ443611	NA	NA	64.90 (72.10)	53.90 (39.10)	53.90 (46.50)
FD53	Dog	GVI.2	UK/2007	JF930689	NA	NA	64.20 (71.20)¶	54.40 (39.10)	54.20 (46.50)
FD210	Dog	GVI.1	Italy /2007	JF939046	NA	NA	65.10 (70.80)¶	54.30 (38.60)	54.20 (44.10)
AN1633	Dog	GVI.1	USA/2017	MK067293	NA	NA	62.60 (48.40)§	55.50 (40.60)	53.10 (43.50)
AN1632	Dog	GVI.1	USA/2017	MK067292	NA	NA	62.40 (47.80)§	55.50 (40.60)	53.10 (43.50)
ITA/91	Dog	GVI.1	Italy /2007	FJ875027	NA	NA	65.10 (71.20)¶	55.00 (39.90)	53.40 (43.80)
63.15	Dog	GVI.2	Italy /2015	KY486329	NA	NA	65.10 (72.10)¶	54.20 (38.80)	55.20 (46.20)
AN1640	Dog	GVI.2	USA/2017	MK067295	NA	NA	62.40 (47.80)§	54.20 (38.90)	54.5 (44.70)
HKU Ca026F	Dog	GVII	China/2007	FJ692500	NA	58.50 (47.20)	62.20 (55.00)	52.90 (37.90)	43.80 (33.00)
HKU Ca035F	Dog	GVII	China/2007	FJ692501	NA	58.50 (47.30)	62.20 (55.00)	52.90 (38.10)	43.80 (33.00)
1C-09	Dog	GII.4	Finland/2009	JF746890	Unclassified	NA	NA	91.60 (91.60)#	NA
261–10	Dog	GII.4	Finland /2010	JF746891	Unclassified	NA	NA	91.60 (91.60)#	NA
3–09	Dog	GII.4	Finland /2009	JF746892	Den Haag 2006b	NA	NA	91.60 (97.40)#	NA
Human									
HuNoV/OC07138	Human	GII.Pe-GII.4	Japan/2007	AB434770	Osaka 2007	NA	94.80 (98.50)**	89.60 (94.60)	99.00 (98.90)
HuNov/NSW001P	Human	GII.Pe-GII.4	USA/2008	GQ845367	New Orleans	89.10 (94.50)	94.50 (86.50)	94.10 (93.90)	93.60 (96.30)
HuNoV/New Orleans	Human	GII.P4-GII.4	USA/2009	GU445325	New Orleans	89.00 (94.70)	94.70 (86.70)	94.30 (93.70)	93.7 (96.10)
HuNoV/NSW0514	Human	GII.Pe-GII.4	Australia/2012	JX459908	Sydney 2012	97.6 (98.70)	98.70 (97.70)	99.20 (97.40)	97.00 (98.00)
HuNoV/CUHK3630	Human	GII.Pe-GII.4	China/2012	KC175323	Sydney 2012	98.20 (99.20)	99.20 (98.20)	99.50 (98.10)	98.00 (98.50)
HuNoV/JN010	Human	GII.Pe-GII.4	China/2017	MG214988	Sydney 2012	99.00 (99.50)	99.50 (99.00)	99.60 (99.00)	98.90 (99.4)
HuNoV/DBM15–156	Human	GII.Pe-GII.4	Thailand/2015	MG786781	Sydney 2012	97.40 (98.80)	97.50 (99.50)§	97.50 (98.50)	95.90 (95.20)
HuNoV /CU21953	Human	GII.Pe-GII.4	Thailand/2018	This study	Sydney 2012	99.90 (99.80)	99.80 (100)	99.90 (100.00)	99.90 (99.80)
HuNoV /CU21954	Human	GII.Pe-GII.4	Thailand/2018	This study	Sydney 2012	99.90 (99.80)	99.80 (100)	99.90 (100.00)	99.90 (99.80)
CaNoV/CU21952	Dog	GII.Pe-GII.4	Thailand/2018	This study	Sydney 2012	99.90 (99.80)	99.80 (100)	99.80 (99.80)	99.9 (99.60)
CaNoV/CU21939	Dog	GII.Pe-GII.4	Thailand/2018	This study	Sydney 2012	100.00 (100.00)	100.00 (100.00)	100.00 (100.00)	100.00 (100.00)

*CaNoV, canine norovirus; HuNoV, human norovirus; NA, not available; ORF, open reading frame; WGS, whole-genome sequencing.
†Genotype classification by the Norovirus Genotype Tool (https://www.rivm.nl/mpf/typingtool/norovir).
‡Norovirus strain NSW0514 (JX459908) was used as a reference. Values are basepairs.
§Size of the ORF1 gene for genetic comparison is 5,088 bp.
¶Size of the ORF1 gene for genetic comparison is 699 bp.
#Size of the ORF2 gene for genetic comparison is 228 bp.
**Size of the ORF1 gene for genetic comparison is 805 bp.

Appendix Table 4. Primers for identification and sequencing of noroviruses, Thailand*

Primer	Sequence, 5′→3′	Position	Target	Reference
F4895	GATTTAGGTGACACTATAGYDSTT YTCHTTYTAYGGKGAYGATGA	4585	RdRp	(*1*)
R5591	AWTCGGGCARGAGATYGCGATC	5078	RdRp	
G2SKR	CCRCCNGCATRHCCRTTRTACAT	5389	VP1	(*2*)
NOV-ORF1–1F	GTGAATGAAGATGGCSTCTAACG	1	ORF1	This study
NOV-ORF1–1R	CCTGTTCCAATCCTGGTACG	705	ORF1	This study
NOV-ORF1–2F	TCTCTCCAGACACTCTTAGG	572	ORF1	This study
NOV-ORF1–2R	GCATCCTCGATGGAYCTCAC	1233	ORF1	This study
NOV-ORF1–3F	TAGGTTTGGTGCTAGGATTTAC	1065	ORF1	This study
NOV-ORF1–3R	CCTTTGTTCTCAATTCTGTC	1740	ORF1	This study
NOV-ORF1–4F	CAGCGYGTRGGYCTTATCC	1580	ORF1	This study
NOV-ORF1–4R	CTGACATRGTCTTGACATCCTT	2208	ORF1	This study
NOV-ORF1–5F	GAGCATCAGGGTTACTCCATG	2066	ORF1	This study
NOV-ORF1–5R	CTCTTGTACTCRTCGTACTCCTCAT	2700	ORF1	This study
NOV-ORF1–6F	CACAGAAGAGATGGCCAACA	2561	ORF1	This study
NOV-ORF1–6R	CTAGAATCATGCCCGTCACATC	3227	ORF1	This study
NOV-ORF1–7F	CTGGTCGCGGATAGTCAACT	3062	ORF1	This study
NOV-ORF1–7R	TTCTTTCCCTCTTCAAACATTAGG	4038	ORF1	This study
NOV-ORF1–8F	TCAARGGTGGCCCTTCATTGC	3726	ORF1	This study
NOV-ORF1–8R	AAGGGAGTTGGCCTGAATGAT	4561	ORF1	This study
NOV-ORF1–9F	CAGAACCACACCTGGCCCAG	4371	ORF1	This study
NOV-ORF1–9R	GTCAATTACATTTTGTTGGCCCGC	5210	ORF1	This study
NOV-ORF2–1F	AGACAAGAGCCAATGTTCAG	5004	ORF2	This study
NOV-ORF2–1R	GTGCCTAGGAGCACGCCATCAG	5887	ORF2	This study
NOV-ORF2–2F	TGAGGAGATGACCAATTCAAGA	5787	ORF2	This study
NOV-ORF2–2R	ATCCAGCAAAGAAAGCTCCAGC	6709	ORF2	This study
NOV-ORF3–1F	AGGTTTGATTCCTGGGTYAACCAG	6630	ORF3	This study
NOV-ORF3–1R	CGTGACTCCCCYCGCTTACG	7487	ORF3	This study
VN3T20	GAGTGACCGCGGCCGCT20		Poly A	(*10*)

*NOV, norovirus; ORF, open reading frame; RdRp, RNA-dependent RNA polymerase; VP, viral protein.

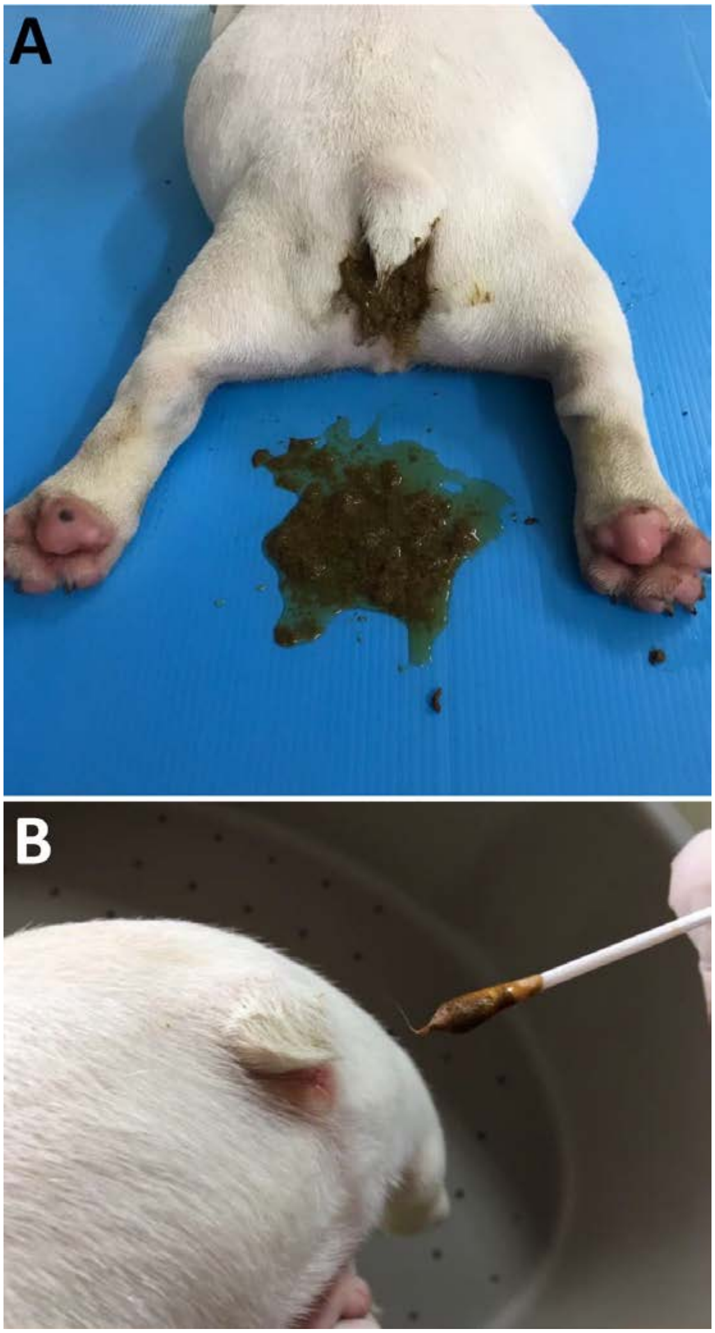

Appendix Figure 1. Human norovirus infection in dogs, Thailand. A) Diarrhea. B) Collection of fecal sample.

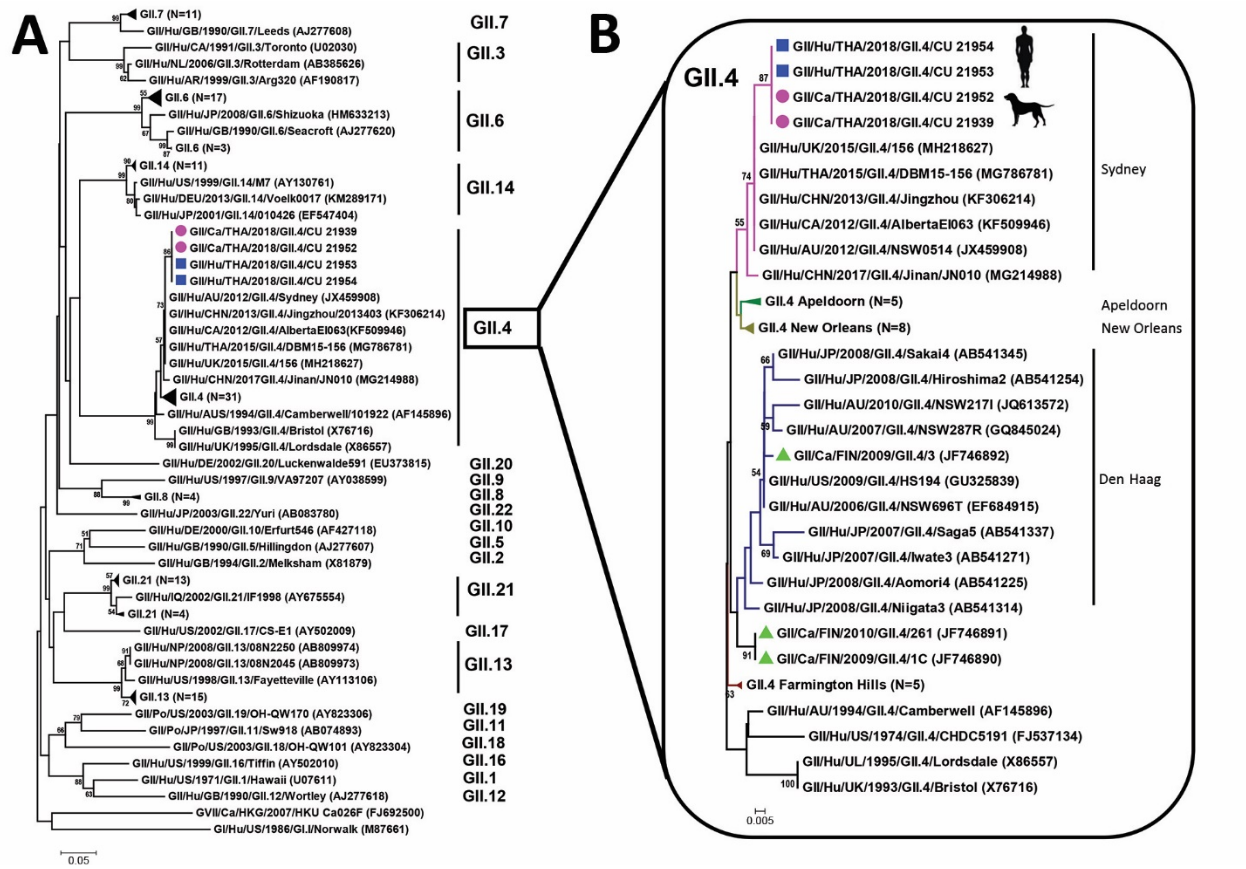

Appendix Figure 2. A) Phylogenetic tree of ORF2 of noroviruses. B) Phylogenetic tree of ORF2 of GII.4 noroviruses. Red circles indicate canine noroviruses from Thailand, green triangles indicate canine noroviruses from Finland, and blue squares indicate human noroviruses from Thailand. Trees were constructed by using MEGA version 7.026 (https://www.megasoftware.net) with the neighbor-joining algorithm and bootstrap analysis with 1,000 replications. Numbers along branches are bootstrap values, and numbers on the right of panel A indicate genogroups. Scale bars indicate nucleotide substitutions per site. ORF, open reading frame.
